# Immune checkpoint inhibitor-induced cholangitis—a three-case series

**DOI:** 10.37349/etat.2024.00250

**Published:** 2024-07-19

**Authors:** Simon Gray, Nuria Santamaria, Anna Olsson-Brown

**Affiliations:** IRCCS Istituto Romagnolo per lo Studio dei Tumori (IRST) “Dino Amadori”, Italy; ^1^Department of Medical Oncology, Clatterbridge Cancer Centre NHS Foundation Trust, L7 8YA Liverpool, UK; ^2^Department of Molecular and Clinical Cancer Medicine, Faculty of Health and Life Sciences, University of Liverpool, L69 7BE Liverpool, UK; ^3^Department of Radiology, Clatterbridge Cancer Centre NHS Foundation Trust, L7 8YA Liverpool, UK

**Keywords:** Immunotherapy, toxicity, cholangitis, immune checkpoint inhibitor, NSCLC, melanoma, renal cell carcinoma

## Abstract

Over the last decade, immune checkpoint inhibitors (ICIs) have dramatically improved the systemic treatment of multiple solid tumour types. However, they can also induce inflammation in an extensive range of normal tissues types. The entity of ICI-induced cholangitis is rare and has not been widely described. We present three cases of ICI-induced cholangitis which illustrate the difficulties associated with its diagnosis and management. We also present associated radiological findings that include intrahepatic duct abnormalities consistent with sclerosing cholangitis-progressive worsening of intrahepatic duct dilatation and pericholecystic haziness suggesting inflammation characteristic of this rare, but severe, toxicity.

## Introduction

Immune checkpoint inhibitors (ICIs) have changed the landscape of systemic anticancer therapy (SACT) over the past decade [1]. Their use has been associated with improved survival in a wide variety of human solid tumours and is increasingly extending beyond the treatment of metastatic disease into adjuvant, and even neoadjuvant settings [2, 3]. The most widely-used ICIs target the programmed death (ligand) 1 and cytotoxic T lymphocyte 4 axes, and may be utilised as monotherapy or in combination. The toxicity profile of ICIs is distinct from that of cytotoxic chemotherapy, insofar as toxicity is less predictable and the range of potential ICI-induced immune-related adverse events (irAEs) is far broader, with the potential to affect almost any organ system. In the absence of higher-quality data, clinical guidance for some of the rarer irAEs remains based on case reports and expert opinion [4]. Grading of irAEs is as per the Common Terminology Criteria for Adverse Events version 5.0 [5].

Any-grade ICI-induced hepatotoxicity (ICI-H) is seen among approximately 5–10% of patients treated with ICI monotherapy (Grade 3: 1–2%) [6, 7]. A subset of patients with more severe ICI-H develop ICI-induced cholangitis (ICI-C), in which liver function test (LFT) abnormalities are predominantly of γ-glutamyltransferase and alkaline phosphatase as opposed to the transaminases, aspartate aminotransferase and alanine aminotransferase [8]. Patients who develop ICI-C typically have ICI-H identified initially, and ICI-C is subsequently diagnosed by ultrasound or cholangiopancreatography, the latter of which may be either endoscopic or magnetic resonance-based (ERCP, MRCP). Three subtypes of ICI-C are described, being typically defined by imaging and in some cases correlated histologically—large duct cholangitis (often termed sclerosing cholangitis), small duct cholangitis, and mixed-type [8]. Treatment of ICI-C is typically with ursodeoxycholic acid (UDCA) to facilitate biliary drainage, and immunosuppression to arrest the ductal inflammation. Corticosteroids are consistently utilised, while additional agents including mycophenolate mofetil (MMF), azathioprine, tacrolimus, and tocilizumab show greater case-to-case variability [8]. We sought to describe three adult cases of ICI-C managed at our centre, including biochemical and radiological findings.

## Case report

### Case 1

**Figure 1:**
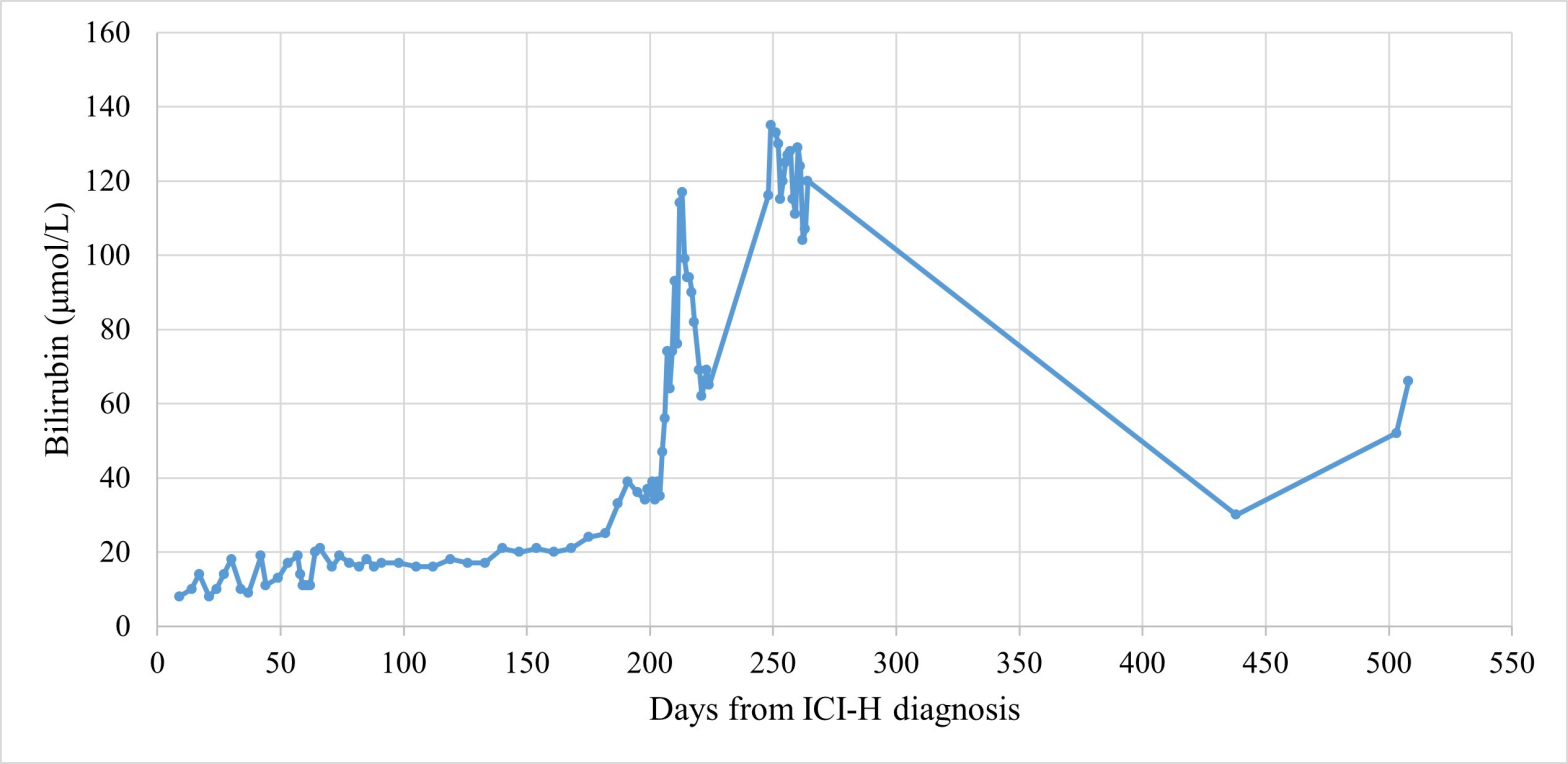
Bilirubin trend for case 1. ICI-H: immune checkpoint inhibitor-induced hepatotoxicity

**Figure 2:**
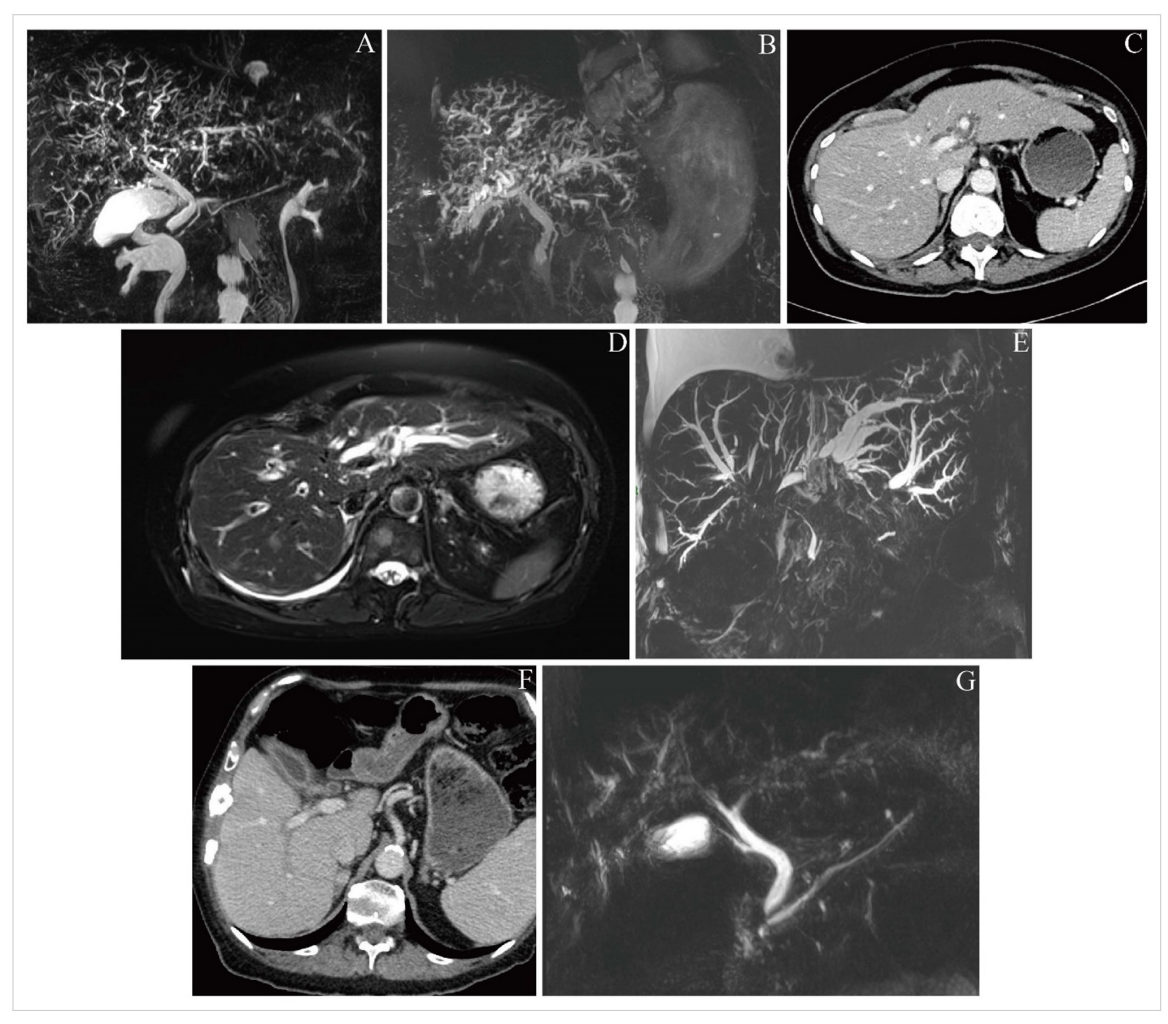
Representative imaging findings for included patients. (A) Case 1; cholangiographic image from magnetic resonance cholangiopancreatography (MRCP) on day 76 after diagnosis of immune checkpoint inhibitor-induced hepatotoxicity (ICI-H), demonstrating intrahepatic duct abnormalities consistent with sclerosing cholangitis; (B) case 1; cholangiographic image from MRCP on day 513, showing stable intrahepatic duct dilatation compared with prior imaging; (C) case 2; computed tomography (CT) image on day 206 after ICI-H diagnosis, demonstrating mild biliary dilatation; (D) case 2; T2 MR image on day 210, demonstrating more pronounced features of sclerosing cholangitis compared with Figure 2C; (E) case 2; cholangiographic image from MRCP on day 217, clearly demonstrating marked intrahepatic biliary dilatation, and a long segment of stenosis within the common bile duct (CBD); (F) case 3; CT image from 15 days before ICI-H diagnosis, demonstrating enhancement of the gallbladder, central bile ducts and CBD; (G) case 3; cholangiographic image from MRCP on day 26 after ICI-H diagnosis, demonstrating the same abnormalities as seen in Figure 2E, as well as intrahepatic duct abnormalities

Patients’ baseline characteristics are displayed in Table 1. Table 2 documents the imaging modalities and treatments utilised, as well as recording LFT results at key time points. After 4 cycles of treatment, the patient was commenced on levothyroxine for ICI-induced hypothyroidism. The patient took no medication for rheumatoid arthritis and was allergic to azathioprine. Routine blood tests showed G3 ICI-H 13 months from initiating treatment and after 8 ICI cycles, at which time the patient attended the hospital acutely and was treated with UDCA and 5 days’ intravenous methylprednisolone (IVMP) at 2 milligrams per kilogram of body weight (mg/kg) once daily, which was converted to a tapering 60 mg prednisolone. Figure 1 charts the patient’s bilirubin throughout their clinical course. On day 4 from ICI-H diagnosis, MR liver showed sludge in the common bile duct (CBD) and gallbladder. Worsening of LFTs was seen on day 13; prednisolone was increased back to 60 mg and MMF commenced [500 mg twice-daily (BD) for 3 days, then increased to 1 g BD]. On day 62, LFTs worsened and prednisolone was changed to IVMP 2 mg/kg for 5 days, followed by 60 mg tapering prednisolone. A small filling defect in the distal CBD and mild dilatation of the intrahepatic bile ducts was reported on the patient’s MRCP (Figure 2A; upon subsequent radiologist review for this case series, evident abnormalities of the intrahepatic bile ducts, consistent with sclerosing cholangitis, were commented on); MMF was increased to 1.5 g BD on day 76. Budesonide was added following further worsening of LFTs on day 94. Multidisciplinary discussion on day 125 advised computed tomography (CT) of the pancreas and endoscopic ultrasound (EUS), which demonstrated sclerosing cholangitis and ongoing intrahepatic ductal inflammation. Tacrolimus 3 mg BD was commenced on day 184, however, due to itching, right upper quadrant pain, and worsening of LFTs, the patient was admitted to hospital and treated with IVMP 4 mg/kg, antibiotics, and antifungals. Liver function tests continued to worsen and *N*-acetylcysteine (NAC) was given over 21 hours, dosed as for paracetamol overdose [9]; following discussions with hepatology, the patient was not considered a liver transplant candidate. A trial of infliximab was suggested, and given (day 213); LFTs stabilised 5 days later. The patient received two further doses of infliximab (days 220 and 249) and was discharged on day 225, but was subsequently re-admitted with worsening hyperbilirubinaemia, jaundice, and encephalopathy, and received palliative care input. There was also concern of a superadded infection with raised C-reactive protein (CRP; 209 mg/L) so antibiotics were given with good biochemical effect (CRP 21 mg/L after a 7-day course). Despite this, ongoing clinical deterioration with worsening LFTs was seen and felt likely to be worsening with inflammatory changes. Given the deterioration and worsening imaging features of sclerosing cholangitis, the prognosis was felt to be poor; after two weeks in hospital, the patient was discharged to hospice and subsequently home with 24-hour care with ongoing MMF. However, over the next several months, the patient gradually improved in terms of symptoms and daily functioning, remained recurrence-free, and demonstrated marked biochemical improvement (Table 2). Despite sustained clinical and biochemical improvement, repeat MR liver at day 513 from ICI-H diagnosis showed stable changes consistent with sclerosing cholangitis versus previous MRCP (Figure 2B).

**Table 1:** Patient baseline characteristics

**Cases**	**Primary disease**	**Sites of cancer at treatment initiation**	**Indication; treatment line**	**Treatment**	**Pre-existing comorbidities**	**Baseline medications**
Case 1	Melanoma (resected stage 3A)	N/A	Adjuvant	Pembrolizumab, 6-weekly	Coeliac disease, osteoarthritis, hiatus hernia, rheumatoid arthritis	Nil
Case 2	Non-small cell lung cancer	Lung, bone, liver, leptomeningeal	Palliative; first-line	Pembrolizumab/Carboplatin/Pemetrexed, 3-weekly	high cholesterol, pulmonary embolus	Atorvastatin, apixaban, paracetamol, levetiracetam
Case 3	Renal cell carcinoma (clear cell)	Lung, bone	Palliative; third-line	Nivolumab, 4-weekly	Type 2 diabetes mellitus, spinal stenosis	Oramorph, zopiclone, lactulose

N/A: not applicable

**Table 2:** The clinical course of immune checkpoint inhibitor-induced cholangitis cases

**Cases**	**Imaging**		**Treatment**						**Timepoint**	**Liver function tests**				
	**US**	**MRCP**	**UDCA**	**Cortico-steroids**	**MMF**	**TAC**	**IFX**	**NAC**		Bil**	**ALP**	**GGT**	**AST**	**ALT**
Case 1			750 mg	4 mg/kg IVMP	1.5 g BD	3 mg BD	5 mg/kg	*	Pre-ICI	5	85	24	20	10
									Day 0	20	981	998	420	389
									MRCP	21	601	862	124	176
									Peak	141	1142	1672	272	352
									Day 482 (most recent)	30	524	1078	76	80
Case 2			-	500 mg IVMP	1 g BD	3 mg BD	-	*	Pre-ICI	12	155	192	51	N/A
									Day 0	5	301	474	44	117
									MRCP	76	808	2249	454	1197
									Peak (and most recent)	453	1165	2785	219	547
Case 3			-	4 mg/kg IVMP	1.25 g BD	3 mg BD	5 mg/kg	-	Pre-ICI	7	147	166	38	50
									Day 0	17	1088	2952	130	183
									MRCP	37	1084	3278	146	247
									Peak	65	1404	4994	262	278
									Day 482 (most recent)	33	974	2559	40	75

Green indicates performed/received, and red indicates not performed/received. The highest doses received for each treatment are specified. Day 0 refers to the day of immune checkpoint inhibitor-induced hepatotoxicity diagnosis, liver function tests are provided for the day of MRCP. Peak values were selected based on the day of the patient’s highest measured bilirubin value. ICI-H: immune checkpoint inhibitor-induced hepatotoxicity; US: ultrasound; MRCP: magnetic resonance cholangiopancreatography; UDCA: ursodeoxycholic acid; mg/kg: milligrams per kilograms of bodyweight; IVMP: intravenous methylprednisolone; MMF: mycophenolate mofetil; BD: twice-daily; TAC: tacrolimus; IFX: infliximab; NAC: *N*-acetylcysteine; Bil: bilirubin; ALP: alkaline phosphatase; GGT: γ-glutamyltransferase; AST: aspartate aminotransferase; ALT: alanine aminotransferase. *: see [9]; **: μmol/L. All other liver function tests in units per litre

### Case 2

This patient had routine blood showing G2 hepatitis 21 days after commencing combination ICI-chemotherapy, having received 1 cycle of ICI and was treated with a 60 mg prednisolone taper. The taper was successfully completed 27 days after ICI-H was diagnosed, however, the patient’s LFTs worsened again on day 33 and a repeat course of prednisolone was given. Response assessment after 3 cycles of ICI-chemotherapy showed treatment response in the lung, some progression in existing bony sites and normal hepatic ducts. Whole-brain radiotherapy was given and the patient had planned to recommence maintenance ICI-chemotherapy but was admitted on day 167. During the admission ICI-induced pneumonitis and malignant pleural effusion were diagnosed, and treated with chest drain and corticosteroids. On day 206 LFTs acutely worsened and the patient underwent a CT showing mild biliary dilatation (Figure 2C) and was treated with 4 mg/kg IVMP, tacrolimus 3 mg BD, and NAC. MR liver on day 210 showed worsening of hepatic metastases and appearances consistent with ICI-C (Figure 2D); accordingly, the patient commenced MMF 500 mg BD, increased IVMP to 500 mg/day, started budesonide 3 mg three times daily, and underwent thiopurine methyltransferase testing and infliximab screening. Dose of MMF was doubled to 1g BD after 3 days. Liver function tests continued to worsen, and visible jaundice developed. Progression of intrahepatic ICI-C appearances, together with stenosis of the CBD, was seen on MRCP on day 217 (Figure 2E). Coagulation became deranged and CT Brain was performed on day 223 for acutely reduced responsiveness, demonstrating a subarachnoid haemorrhage. The focus of management was changed to end-of-life care, and the patient died on day 225.

### Case 3

This patient had previously received tivozanib and cabozantinib and commenced palliative ICI monotherapy. They presented with ICI-H 25 weeks after ICI initiation having received 3 ICI cycles (treatment delays were due to hypercalcaemia requiring hospital admission). A routine CT scan 15 days before ICI-H was reported as showing pericholecystic haziness suggesting inflammation (Figure 2F; upon subsequent radiologist review for this case series, enhancement of the gallbladder, central bile ducts, and CBD were commented on). The patient commenced 60 mg tapering prednisolone without improvement of LFTs. On day 29, MRCP showed dilated right peripheral bile ducts suggesting ICI-C (Figure 2G); the patient was accordingly commenced on MMF and 2 mg/kg IVMP, which was later (day 44) increased to 4 mg/kg and tacrolimus also commenced due to lack of LFT response. After 9 days of IVMP (day 51), 60 mg tapering prednisolone was commenced with MMF increased to 1.25 g BD. On day 58, troponin T and amino-terminal pro-B-type natriuretic peptide (NTproBNP) values were raised acutely (71 ng/mL and 899 pg/mL respectively); ICI-induced myocarditis was suspected and 4 mg/kg IVMP started. Infliximab was given (day 62) and IVMP was continued until cardiac MR was performed (day 76); it demonstrated ischaemia and a previous infarct, but no changes of ICI-induced myocarditis. Accordingly, IVMP was converted to tapering prednisolone, though a second dose of infliximab was given (day 97) due to worsening of LFTs. Liver function tests all began to normalise following treatment (Table 2) and immunosuppression was de-escalated. Ongoing endocrine input was required for malignancy-associated hypercalcaemia and the patient experienced two subsequent admissions for this (days 113 and 125). During the second admission, the patient developed a Covid-19 infection and unfortunately died as a result of this on day 161.

## Discussion

The cases described here illustrate some of the heterogeneity seen in cases of ICI-C, in terms of severity, biochemical onset, radiological findings, requirements for treatment and clinical outcome. Many cases reported in the literature report immunosuppressant medication use as present/absent, whereas here specific doses of medications received are specified, as is the chronology of their use which is useful, especially given the relapsing and remitting nature of many cases of ICI-C [8, 10]. The inclusion of radiologic images that can be aligned with pharmacological management is also a strength of the present series [8, 10].

Diagnosis of ICI-H can be challenging in patients with known liver metastases, or concurrently receiving other potentially hepatotoxic SACT. The temporal pattern of a patient’s LFTs is likely to be useful: chemotherapy, unlike ICIs, is likely to produce a transient derangement which resolves on holding the offending agent; case 2 in the present series illustrates this, with baseline liver metastases and treatment with ICI-chemotherapy. Case 2 also illustrates the potential severity of ICI-C, with rapidly progressive radiological appearances (Figure 2C–E) alongside gross hyperbilirubinaemia (peak value 453 μmol/L) and coagulopathy (1.4-fold increase in prothrombin time versus baseline) contributing to subarachnoid haemorrhage, and subsequently death.

Case 1 is notable for its severity and refractoriness to multiple treatments, with apparent stabilisation following infliximab therapy; this finding warrants subsequent exploration particularly as guidance from the European Society of Medical Oncology (ESMO) advises avoiding infliximab in ICI-H due to a possible association with idiosyncratic liver failure, though this has not been observed in the context of irAEs [4]. Indeed, ESMO guidance only specifically recommends UDCA and corticosteroids in ICI-C [4]; the American Gastroenterological Association advises considering infliximab for severe ICI-H on a case-by-case basis with no specific mention of ICI-C, while American Society of Clinical Oncology guidelines state infliximab is contraindicated in ICI-H [11, 12]. These cases also illustrate our institutional experience that for patients whose ICI-C is treated with initial success, the course is typically prolonged, requires multiple immunosuppressant medications, and attempting to taper these prematurely commonly leads to flaring of toxicity. We include our centre’s local guidance for diagnosis and management of ICI-H as supplementary materials.

Regarding the radiological findings of ICI-C, we note that in several of the cases described, rather than requiring MR to detect any signs of ICI-C, relevant abnormalities were detected using CT imaging in the first instance, which prompted MR for a more complete evaluation. Specifically, CT imaging displayed CBD enhancement in case 1, as well as enhancement of the CBD and gallbladder in case 3 (Figure 2E). Previous comparisons of imaging in pancreatobiliary disease have demonstrated the added value of MR for diagnosis, particularly in the evaluation of the extrahepatic bile ducts [13]. As radiologists often only have access to the clinical information provided on imaging requests rather than a full clinical history, clinicians should be aware to include details of ICI treatment and possible cholangitis on requests for CT, as well as MR imaging, if the clinical picture is consistent. Increased awareness of ICI-C among radiologists, particularly among those who do not specialise in hepatobiliary radiology, will further help to optimise the diagnostic process. We report that partially due to the cases described herein, our centre’s protocols for MR liver scans in patients who have received ICI were altered to include some sequences previously reserved for MRCP, to improve diagnosis of ICI-C at our centre. In the future, radiomics may assist in the rapid assessment of tumour response which could facilitate early cessation of therapies in patients who are not benefiting, which may help to prevent treatment-related toxicities in the metastatic and neoadjuvant settings [14, 15].

In this series, none of the patients underwent liver biopsy during their ICI-C management. Biopsy findings of cases reported elsewhere [8] have been demonstrated to vary depending on subtype—large-duct versus small-duct. There is currently no evidence for managing these subgroups differently, and so a biopsy is not routinely pursued at our centre. However, developing more optimal management approaches is likely to depend on a deeper understanding of the biochemical, radiological, and histological subtypes of ICI-C, and for this reason, prospective clinical trials including liver biopsy in a research context should be advocated for.

In summary, ICI-C is rare and severe toxicity that can present with varying tempo, provides characteristic findings on imaging, and often requires a complex immunosuppressive approach with tumour necrosis factor alpha-inhibiting agents illustrating therapeutic benefit.

## Abbreviations

BD: twice-daily
CBD: common bile duct
CT: computed tomography
ICI-C: immune checkpoint inhibitor-induced cholangitis
ICI-H: immune checkpoint inhibitor-induced hepatotoxicity
ICIs: immune checkpoint inhibitors
irAEs: immune-related adverse events
IVMP: intravenous methylprednisolone
MMF: mycophenolate mofetil
MRCP: magnetic resonance cholangiopancreatography
UDCA: ursodeoxycholic acid

## Supplementary materials

The supplementary material for this article is available at: https://www.explorationpub.com/uploads/Article/file/1002250_sup_1.pdf.

## Declarations

### Availability of data and materials

Partial raw data (clinical records including documentation and imaging reports) for this manuscript are not publicly available because data contains personally identifiable information that may be restricted from public access to protect individuals’ privacy.

### Copyright

© The author(s) 2024.
