# Supplementary material for: Immune checkpoint inhibitor-induced cholangitis—a three-case series
Source: Explor Target Antitumor Ther. 2024 Jul 19;5(4):818–25. doi: 10.37349/etat.2024.00250 (PMC11390286; doi:10.37349/etat.2024.00250)
Supplement: Supplementary file 1 [file 1002250_sup_1.pdf]

The Clatterbridge Cancer Centre
NHS Foundation Trust

Immune-Related Adverse Event Guideline: Hepatoxicity

Hepatic transaminases (ALT/AST) and bilirubin must be evaluated before each dose of immunotherapy, as early laboratory changes may indicate emerging immune-related hepatitis. Elevations in LFTs may develop in the absence of clinical symptoms. This guidance should be used in context of baseline LFTs and presence of known liver metastases. No dose adjustment is required for mild hepatic impairment but data is limited for use of these drugs in moderate/severe hepatic impairment and patients should be closely monitored for elevation in LFTs from baseline.
Prior to commencement of immunotherapy all patients should have LFTs checked

Mild (Grade 1)

AST or ALT < 3 x ULN but increasing from baseline

Investigations:
- Weekly LFT (including ALT) check between cycles of immunotherapy and ensure remain stable prior to next cycle. Inform oncology team
- Consider culprit concomitant medications

Actions:
- Continue immunotherapy treatment

Biochemical Abnormality WORSENS or RELAPSE see moderate/ severe strand (LFT dependant)

Moderate (Grade 2)

AST or ALT >3 to ≤5 x ULN

Clinical Assessment
As per mild (grade 1) +

Investigations:
- Regular LFTs, direct and indirect bilirubin and clotting profile
- MRI/USS of liver to exclude PD & thromboembolism and evaluate if evidence of inflammation
- Hepatitis viral panel (hepatitis A, B, C, E)
- CMV, EBV and HIV and auto-antibodies

Treatment:
- Commence prednisolone 60mg/day+ gastric protection

Actions:
- Withhold dose until the adverse reaction resolves to Grade 0-1 (or returns to baseline).
- Review medications (e.g. statins, antibiotics)
- **Re-check LFTs and INR every 3 days and review patient by phone twice weekly. If improving check LFTs weekly.**

Symptoms: Resolve or Improve to Mild See steroid tapering guidance

Biochemical Abnormality PERSISTS (≥3 days), WORSEN or RELAPSE see severe strand

Severe or Life-Threatening (Grade 3/4)

AST or ALT >5 x ULN (Grade 4 >20 x ULN)

Clinical Admission
As per moderate (Grade 2) +
Consider admission of patient

Investigations:
- Daily LFTs, clotting profile and lactate. If deteriorating, consider venous gas.
- MRI of liver to exclude PD & thromboembolism and evaluate if evidence of inflammation or sclerosing cholangitis
- Exclude other causes (e.g. Heart failure/ PD)

Treatment:
- IV methylprednisolone 2mg/kg/day
- Increasing to 4mg/kg/day could be considered if clinical improvement is unsatisfactory.
- IV hydration (patients need to be well hydrated to promote hepatic perfusion with fluid balance)
- Vitamin K 10mg IV daily x 3 days if INR deranged
- **Grade 4 (loss of synthetic function or hyperbilirubinemia) commence**

N-acetylcystine (NAC as per paracetamol overdose protocol in BNF)
If albumin low, discuss with hepatologist and consider administration of human albumin solution (HAS)

Actions:
- Referral to hepatologists for further advice
- Consider antibiotic prophylaxis with patients on high dose, prolonged steroids
- Establish escalation plan and ceiling of care

Symptoms: Resolve or Improve to Mild See steroid tapering guidance

Assess response to treatment, if no improvement within 72 hours, consider additional immunosuppression. Consider CCC Subsequent Management guidelines

Abbreviations
LFTs = liver function tests

INR = international normalised ratio
ULN = upper limit of normal
PD = progressive disease

Interrupt SACT immunotherapy until discussed with Acute Oncology Team. Please contact on-call oncology/haematology team for advice. Ensure that the patient has monitoring/follow up planned with their oncology/immuno-oncology team.

**** IMPORTANT **Subsequent management of patients with hepatitis that is either not improving or worsening should always be discussed with a gastroenterologist and the patient's oncologist**

Subsequent management guideline– Hepatitis

Outpatient management

Following on from Initial Management Guidelines or Discharge

→ To commence oral prednisolone 60mg/day and taper 10mg prednisolone every 5 days with bi-weekly telephone monitoring and LFT's.
Referral to the IO team to commence outpatient toxicity follow up [redacted]

Assess LFTs and clinical status

- **Improving** → Extend to weekly calls and bloods after 2 weeks until completion of prednisolone taper as per steroid tapering guidelines. Follow outpatient follow up guidelines
- **LFT's stable/deterioration (admission not indicated)** → Increase to preceding dose of prednisolone (ie. If now on 20mg and LFT's deteriorate, increase to 30mg daily) contact the oncology team (CNS/ANP/medic) identified lead contacts for each tumour group. Increase frequency of monitoring. If do not improve team contact to consider discussion with hepatologist and admission.
- **LFT's deteriorating (admission indicated)** → Admit and start IV methylprednisolone 2mg/kg/day. Follow Initial Management guidelines for Hepatitis for the first 24-48 hours. Discuss ceiling of care with oncology team. Inform heptatology team (normally Dr [redacted] team at [redacted]) of admission

Inpatient Management

Following on from Inital Management Guidelines

Day 3

If LFTs/clinically worsening- Contact Dr [redacted] or local hepatology team for advice consider additional immune-suppression (options include MMF or tacrolimus). Consider increase methylprednisolone to 4mg/kg/day. Continue NAC, IV steroids, IV fluids (depending on fluid status)

Day 5

- **Bloods & clinical status worsened** → **Discuss with Dr [redacted]/ Heptatology team.** Methylprednisolone can be increased but should **NEVER** be increased further without prior agreement from Hepatology as the need to increase signifies steroid resistance and additional immunosuppression may be started in addition or as alternative (options include MMF or tacrolimus). Discuss transfer out to tertiary liver centre.Check results of all investigations.
- **Bloods & clinical status improved** → [a]If IV methylprednisolone > 2mg/kg/day reverse sequence of escalation every 3 days, once down to 2mg/kg/day convert to oral prednisolone 1mg/kg/day (not a flat dose of 60mg).
Follow outpatient subsequent management pathway: bi weekly bloods and taper 10mg every 5 days as **per outpatient pathway[a].**
If on additional immune-suppression, continue this at the same dose whilst the steroids are being reduced, once prednisolone discontinued, wean off immunosuppression as long as LFT's remain stable.

ADDITIONAL IMMUNOSUPRESSION ONLY COMMENCE FOLLOWING DICUSSION WITH THE HEPATOLOGY TEAM

Tacrolimus
-Starting Dose-3mg then level dependant. Discuss with hepatology team (Dr [redacted] team at [redacted]), if level out of range.
-Therapeutic trough levels 7-10
-Trough Tac levels daily during initiation (blood levels to be taken and sent to [redacted] dose to be given immediately after).
- Next dose to be based upon level. Once stable levels not necessary

Mycophenylate Mofitil
Starting dose is 500mg BD increasing to 1g BD after a week
Only monitoring is regular FBC
No levels required

Stopping immunosupression
-Once LFTs improving reduce steroids using the reduction schedule here[a] and then standard weaning protocol.
-Continue immunosuppression until steroids weaned. If LFTs stable for one (1) further week after stopping steroids stop immunosuppression

Dr [redacted] at [redacted] is the regional hepatology advisor for CPI-induced hepatitis and may be contacted by local teams for discussion/advice
